# Impact of COVID-19 lockdown on physical activity behavior among students in Somalia

**DOI:** 10.3934/publichealth.2024023

**Published:** 2024-04-10

**Authors:** Sameer Badri Al-Mhanna, Alexios Batrakoulis, Abdulrahman M. Sheikh, Abdulaziz A. Aldayel, Abdulwali Sabo, Mahaneem Mohamed, Hafeez Abiola Afolabi, Abdirizak Yusuf Ahmed, Sahra Isse Mohamed, Mehmet Gülü, Wan Syaheedah Wan Ghazali

**Affiliations:** 1 Center for Global Health Research, Saveetha Medical College and Hospitals, Saveetha Institute of Medical and Technical Sciences, Saveetha University, Tamil Nadu, India; 2 Department of Physiology, School of Medical Sciences, Universiti Sains Malaysia, Kubang Kerian, Kelantan, Malaysia; 3 Department of Physical Education and Sport Science, School of Physical Education, Sport Science and Dietetics, University of Thessaly, Trikala, Greece; 4 Department of Physical Education and Sport Science, School of Physical Education and Sport Science, Democritus University of Thrace, Komotini, Greece; 5 Faculty of Health Science, Somali International University, Mogadishu, Somalia; 6 Department of Exercise Physiology, King Saud University, Riyadh, Saudi Arabia; 7 Department of Public and Environmental Health, Faculty of Basic Medical Sciences, College of Medicine and Allied Medical Sciences, Federal University Dutse, Dutse, Nigeria; 8 Department of General Surgery, School of Medical Sciences, Hospital Universiti Sains Malaysia, University Sains Malaysia, Kubang Kerian, Kelantan, Malaysia; 9 Demartino Hospital, Ministry of Health, Mogadishu, Somalia; 10 National Public Health Reference Laboratory, Ministry of Health, Mogadishu, Somalia; 11 Department of Sports Management, Faculty of Sport Sciences, Kirikkale University, Kirikkale, Turkey

**Keywords:** coronavirus diseases, facilitators, lifestyle, exercise, well-being

## Abstract

**Background:**

Due to the worldwide reach of the COVID-19 pandemic, authorities across the globe deemed it essential to enforce exceptional containment measures. Maintaining physical activity (PA) during this time was only feasible through engaging in activities at home. Therefore, this study focused on elucidating the levels of PA and well-being among Somali students in the aftermath of the lockdown measures implemented by governments at the onset of the COVID-19 pandemic.

**Methods:**

This study was conducted in Somalia among undergraduate students studying at Somali International University. A total of 1266 students were included in the present study. An online survey was utilized to measure participant PA behavior. The assessment of PA was conducted in the aftermath of the COVID-19 pandemic, utilizing the Godin Leisure questionnaire. The study showed that 85.8% of the study participants (n = 1086) were between the ages of 17 and 22. More than half of the participants (58.7%, n = 743) were female and had no other employment (57.3%, n = 743).

**Results:**

Jogging was the most frequently reported PA (57.3%, n = 726), and PA level was on average 59.7 minutes per day (SD = 25.9). Also, most of the study participants were in their last year (82.1%). In the regression analysis, age, gender, academic year, and work status were significant predictors of being physically active after the COVID-19 pandemic.

**Conclusion:**

Factors affecting PA after the COVID-19 pandemic include age, gender, academic year, and work status. Males, younger individuals, and those who engage in outdoor exercise are more likely to be physically active. Once the COVID-19 restrictions were relaxed, undergraduate students in Somalia were physically active. A high level of PA appears to be advantageous for public health. Universities in Somalia should uphold school policies that promote an active lifestyle among students, aiming to maintain or enhance the existing level of PA.

## 1. Introduction

A health threat and several episodes of pneumonia were blamed in China in December 2019 on a unique coronavirus strain. The World Health Organization classified the disease as coronavirus disease (COVID-19) and defined it as a pandemic [1]. Globally, there were over 800,000 confirmed positive cases by April 2020, and more than 40,000 people died as a result [2]. More than 200,000 people had died from COVID-19 by the middle of 2020, with an estimated 2.9 million infected in more than 200 countries [3]. Until January 25, 2021, the global tally recorded over 99 million cases and 2.1 million fatalities [4].

During the pandemic, international restrictions were implemented to lessen socialization and viral transmissions. These policies implemented by the Somalian administrations had a negative impact on people's social well-being, particularly students [5]. The restrictions placed on students made it evident that their physical activity (PA) behavior would suffer adverse effects both during and after the pandemic, as a result of the limitations imposed by the suspension of all sporting and educational events, including access to recreational facilities; also, the students were vulnerable to psychological exhaustion and restricted PA [6]–[9]. These setbacks resulted in an imbalance in students' concentration, PA, motivated behavior status, and mental health [10],[11]. According to Brooks Webster Smith et al. [12], the negative psychological consequences of the COVID-19 pandemic and quarantine included stress syndrome, anxiety, and depression, along with experiences of social isolation, loneliness, and limitations on travel. These implications may have had an impact on students' well-being. As a result of these concerns, institution officials made ensuring the safety of students on and off campus the highest priority, as the unanticipated COVID-19 effects would have an impact on their physical health and ability to concentrate in school [13].

Hence, students were encouraged to uphold the established control and preventive measures mandated by the government and institutional authorities. This was to ensure the continuation of indoor physical activities, safeguard their health, and prevent the transmission of COVID-19 [14]. Interestingly, digital fitness services, online programs, exercise apps, and home-based training solutions have been reported as popular health and fitness trends during the COVID-19 lockdown [15]–[17]. Collectively, exercise has been shown to lower psychiatric problems, enhance health, and lower the chance of developing severe respiratory distress syndrome [18]. Regular PA is influenced by various factors including gender, smoking status, and place of residence (rural or urban) [19]–[22]. Regular participation in moderate PA is closely linked to health and well-being, and individuals who engage in such activities are prone to experiencing reduced levels of stress, worry, and anxiety [23],[24]. In most nations, the COVID-19 pandemic mentally affected patients, adolescents, elderly individuals, and college students [25]–[28]. Somalia implemented a nationwide lockdown during the COVID-19 pandemic on 16 March 2020; after the relaxation of the COVID-19 lockdown, the study was conducted.

Nevertheless, there is currently no documented research on the well-being and PA patterns of Somali students in the aftermath of the COVID-19 pandemic [29]. Therefore, the main objective of this study is to outline the PA and well-being status of Somali students in the aftermath of the six-month lockdown implemented at the onset of the pandemic. Furthermore, we aimed to pinpoint variations in PA engagement, obstacles, and support factors among active and inactive students in relation to pre-lockdown conditions. Additionally, we explored the impact of students' well-being on the distinctions between indoor and outdoor PA. The study was conducted in Somalia among undergraduate students studying at Somali International University

## 2. Methods

### 2.1. Study population and sample

The study included Somali students studying at Somali International University, with a student population of approximately 8999 students undertaking various degree programs. G*Power software 3.1.9.2 was used to determine the sample size for this investigation. The sample size was set at a maximum of 128, taking into account a type I error of 0.05, a power of 80%, a group size of 2, and a medium effect size of 0.25. The sample size was determined based on a correlation of 0.5 between the repeated readings and a non-sphericity correction ε of 1. A total of 1266 students were included in the present study. All recruited students completed the permission form and voluntarily consented to participate in the research. The investigation encompassed all undergraduate students who engaged with the social media groups affiliated with the institution. The study did not include postgraduate students. The research obtained ethical approval by the Somali International University with the reference number REF:/SIU/RE2002/0002.

### 2.2. Measures

In this cross-sectional study, validated online questionnaires were utilized [30] to gather data in the English language; no translations were performed. All university students studied in English. The participants responded to the online survey by clicking on a link provided on their personal social media platform. The surveys employed in this study were encoded to ensure the confidentiality of the participants. Data were collected between July 2021 and January 2022.

#### 2.2.1. PA behavior

The assessment of PA was conducted in the aftermath of the COVID-19 pandemic, utilizing the Godin Leisure questionnaire. This was done to examine the present level of participants' PA and categorize them as either “active” or “inactive” based on the recorded engagement in “vigorous”, “moderate”, and “light” PA [31]. Drawing on the PA guidelines delineated in the Godin Leisure questionnaire for health-related benefits, individuals were considered active if they engaged in more than 150 min of moderate-to-vigorous PA per week. Conversely, those who fell below the threshold (≤149.9) were classified as inactive [32]. These classifications were employed to categorize individuals per their PA levels.

Additional questions about PA were posed to assess the present PA patterns. This delved into the predominant type of PA, its setting (indoor, outdoor, or both), and whether any changes in location occurred due to social distancing measures. Furthermore, it explored whether there were alterations in PA levels (same, more, or less) since the implementation of restrictions compared with the pre-COVID-19 era. To analyze potential hindrances and support factors for PA benefits, enjoyment, confidence, and readiness for PA engagement, the responses were categorized using three Likert scales: more active, unchanged, and less active. Additional questions were employed to explore the potential impact of social distance on difficulties, support, and opportunities for PA engagement.

#### 2.2.2. Outdoor physical activity

The students reported on the significance of the activity, whether it occurred in a natural environment, and the duration of time spent engaging in outdoor PA each day. Moreover, supplementary components from the scale measuring connectedness to nature were incorporated based on their impact on PA behavior [33]. The comprehensive questions on nature-relatedness assess an individual's emotional, cognitive, and physical bond with the natural environment. The measurement of one's connection to nature is obtained by rating each item on a Likert scale ranging from 1 (strongly disagree) to 5 (strongly agree). The scale measuring one's connection to nature has been linked to behavior, the surrounding environment, and the frequency of individuals engaging in nature-based activities. On a rating scale from 1 to 10, participants were also asked to assess how they were connected to nature [34].

### 2.3. Statistical analysis

Descriptive analysis and inferential statistics were the types of statistical analyses used in the current study. The mean, standard deviation, frequency, and percentages are displayed in the descriptive analysis. One-way analysis of variance (ANOVA), the chi-square test, and binary logistic regression analysis were the inferential statistics. The nature, barriers, and facilitators of PA and well-being across the categories of PA participation were examined using an ANOVA to estimate the mean difference (i.e., much, same, and less active). The association between the participants' characteristics and their participation in PA was examined using the chi-square test. The binary logistic regression analysis was carried out to determine important predictors for engaging in physical exercise. After performing a simple logistic regression to get the predictors' crude odds ratios (CORs), those with p-values of less than 0.25 were deemed significant predictors and included in a multiple logistic regression to determine the predictors' adjusted odds ratios (AOR). Multiple logistic regression was performed using the forward LR and back LR methods, and the final model was generated using the enter method. SPSS version 27 was used for all statistical analyses.

## 3. Results

Table 1 shows that 85.8% of the study participants (n = 1086) were between the ages of 17 and 22. More than half of the participants (58.7%, n = 743) were female and had no other employment (57.3%, n = 743). Jogging was the most frequently reported PA (57.3%, n = 726), and PA level was on average 59.7 minutes per day (SD = 25.9). Also, most of the study participants were in their last year (82.1%); the survey's distribution through an online questionnaire aimed at voluntary participants might have resulted in a higher proportion of respondents from year 4, given that the majority of the surveyed population consisted of students from this group. This is because of factors such as enrolment trends, retention rates, and particular university program structures. In the regression analysis, age, gender, academic year, and work status were found to be significant predictors of being physically active after the COVID-19 pandemic. The 17–22-year-old students were 2.23 times more likely to be physically active than the 23–28-year-old students (AOR = 2.23, p < 0.001). The males were 30% more likely to be physically active than the females (AOR = 1.30, p = 0.042). For the academic year, the year-1 students were 90% less likely to be physically active than the year-4 students (AOR = 0.10, p = 0.024); the year-2 students were 50% less likely to be physically active than the year-4 students (AOR = 0.50, p = 0.023); and the year-3 students were 71% less likely to be physically active than the year-4 students (AOR = 0.29, p < 0.001). For work status, those with no work were 3.17 times more likely to be physically active than the laid-off (AOR = 3.17, p = 0.003); those with reduced hours were 4.39 times more likely to be physically active than the laid-off (AOR = 4.39, p < 0.001); and those with remote work were 8.11 times more likely to be physically active than the laid off (AOR = 8.11, p < 0.001).

Those who reported exercising outside (36.1%) were substantially more active than students who reported exercising inside (14.4%; p < 0.001) (Table 2). Physical activity was much higher among those who thought the environment was very important (36.8%; p < 0.001) (Table 3). For nature-related questions, “enjoying digging in the earth and getting dirt on my hands” and “connectedness to nature on the scale” significantly affected PA participation (p < 0.05). In the barriers and facilitators to PA engagement questions, the support received for exercising regularly and the opportunities for exercising regularly had a significant effect on PA participation (p < 0.05).

For well-being outcomes, “not being able to stop or control worrying”, worrying too much, being easily annoyed or irritable about different things, “feeling afraid as if something awful might happen”, “people seeing me as loving and friendly”, and not experiencing trusting relationships with others had a significant effect on PA participation (p < 0.05) (Table 4).

**Table 1.** Participants' characteristics.

**Variables**	**F (%)**	**Mean (SD)**
Age (years)		
17–22	1086 (85.8)	
23–28	180 (14.2)	
Gender		
Male	523 (41.3)	
Female	743 (58.7)	
Work status		
No work	725 (57.3)	
Reduced hour	415 (32.8)	
Remote work	64 (5.1)	
Laid off	62 (4.9)	
The most common type of physical activity	
Jogging	726 (57.3)	
Games	305 (24.1)	
Yoga	47 (3.7)	
Gym	179 (14.1)	
None	9 (0.7)	
Total PA (minutes)		59.7 (25.9)
Physical activity since the social distance	
More	385 (30.4)	
Same	259 (20.5)	
Less	622 (49.1)	
Academic year		
First	17 (1.3)	
Second	79 (6.2)	
Third	130 (10.3)	
Fourth	1040 (82.1)	

**Table 2.** Correlation of factors associated with physical activity engagement post-COVID-19.

**Variables**	**COR (95% CI)**	**p-value**	**AOR (95% CI)**	**p-value**
Age				
17–22	2.32 (1.55, 3.49)	<0.001	2.23 (1.43, 3.46)	<0.001
23–28	1		1	
Gender				
Male	1.44 (1.13, 1.84)	0.003	1.30 (1.01, 1.68)	0.042
Female	1		1	
Academic year
First	0.12 (0.02, 0.94)	0.043	0.10 (0.01, 0.74)	0.024
Second	0.54 (0.31, 0.94)	0.030	0.50 (0.28, 0.91)	0.023
Third	0.32 (0.19, 0.53)	<0.001	0.29 (0.17, 0.50)	<0.001
Fourth	1		1	
Work status				
No work	2.96 (1.39, 6.32)	0.005	3.17 (1.48, 6.81)	0.003
Reduced hour	2.88 (1.33, 6.22)	0.007	4.39 (2.00, 9.62)	<0.001
Remote work	6.75 (2.77, 16.43)	<0.001	8.11 (3.25, 20.23)	<0.001
Laid off	1			

Note: CI = confidence interval, COR = crudes odds ratio, AOR = adjusted odds ratio.

**Table 3.** Associative changes in participants' physical activity behavior post-COVID-19.

**Participants' characteristics**	**Physical activity participation**			
	**More ** **n (%)**	**Same ** **n (%)**	**Less ** **n (%)**	**p-value**
Where do you engage in PA				<0.001
Indoor	69 (17.4)	58 (14.6)	269 (67.9)	
Outdoor	185 (36.1)	104 (20.3)	223 (43.6)	
None	130 (37.6)	93 (26.9)	123 (35.5)	
Is it a natural environment?				0.500
Yes	307 (29.7)	214 (20.7)	513 (49.6)	
No	78 (33.6)	45 (19.4)	109 (47.0)	
Is the environment necessary in your chosen PA?				<0.001
Extremely important	94 (24.3)	70 (18.1)	223 (57.6)	
Very important	184 (36.8)	102 (20.4)	214 (42.8)	
Somewhat important	72 (28.7)	54 (21.5)	125 (49.8)	
Not so important	28 (32.9)	19 (22.4)	38 (44.7)	
Not at all important	7 (16.3)	14 (32.6)	22 (51.2)	
How essential is nature in your chosen PA?				0.193
Extremely important	103 (29.8)	64 (18.5)	179 (51.7)	
Very important	152 (31.8)	91 (19.0)	235 (49.2)	
Somewhat important	81 (33.3)	55 (22.6)	107 (44.0)	
Not so important	27 (22.3)	27 (22.3)	67 (55.4)	
Not at all important	22 (28.2)	22 (28.2)	34 (43.6)	

**Table 4.** Participants' responses to questions post-COVID-19.

**Questions**	**More ** **(n = 385)**	**Same ** **(n = 259)**	**Less ** **(n = 622)**	**p-value**
**Nature-related questions**	
“I enjoy being outdoors, even in unpleasant weather”	2.94±1.32	2.85±1.32	2.84±1.46	0.493
“My ideal vacation spot would be a remote wideness area”	2.76±1.17	2.73±1.18	2.85±1.17	0.300
“I enjoy digging in the earth and getting dirt on my hands”	2.35±1.14	2.21±1.14	2.50±1.32	0.009
“I take notice of wildlife wherever I am”	2.77±1.13	2.89±1.07	2.91±1.14	0.120
“I don't often go out in nature”	3.34±1.20	3.21±1.18	3.29±1.14	0.370
“The thought of being deep in the woods away from civilization is frightening”	3.20±1.09	3.27±1.11	3.16±1.06	0.339
“Connectedness to nature on the scale number”	6.03±2.48	5.48±2.85	5.82±2.56	0.003
**Barriers and facilitators to physical activity engagement**			
“How beneficial is it for you to exercise regularly right now?”	3.11±1.33	2.98±1.32	3.18±1.18	0.117
“How enjoyable is it for you to exercise regularly right now?”	3.00±1.28	3.12±1.20	3.05±1.15	0.466
“How difficult is it for you to exercise regularly right now?”	2.64±1.13	2.46±1.15	2.62±1.16	0.112
“How confident are you that you can exercise regularly right now?”	2.69±1.29	2.63±1.31	2.75±1.26	0.374
“How motivated are you to exercise regularly right now?”	3.02±1.18	3.05±1.25	2.99±1.14	0.729
“How detailed of a plan do you have for exercising regularly right now?”	2.66±1.03	2.75±1.14	2.76±1.14	0.320
“Have you found it more challenging to engage in regular exercise due to social distance?”	2.81±1.19	2.67±1.23	2.76±1.13	0.349
“How much support do you have for exercising regularly right now?”	2.38±1.07	2.47±0.96	2.61±1.02	0.003
“How many opportunities do you have for exercising regularly right now?”	2.43±1.01	2.48±0.86	2.66±0.97	0.001
**Well-being outcomes**	
“Being so restless that it is hard to sit still”	2.57±0.87	2.62±0.96	2.59±0.94	0.792
“Feeling nervous, anxious or on edge”	2.08±0.89	1.97±0.84	2.11±0.85	0.085
“Not being able to stop or control worrying”	2.20±0.87	2.00±0.90	2.00±0.85	0.001
“Worrying too much”	2.19±0.87	1.92±0.86	2.05±0.84	<0.001
“Trouble relaxing much about different things”	2.11±0.89	2.03±0.85	2.08±0.84	0.581
“Becoming easily annoyed or irritable about different things”	2.04±0.84	1.85±0.81	2.07±0.80	0.001
“Feeling afraid, as if something awful might happen”	1.89±0.87	1.90±0.86	2.02±0.88	0.032
“Most people see me as loving and affectionate”	4.02±2.11	4.13±2.13	4.44±2.08	0.006
“Maintaining close relationships has been difficult”	3.20±1.20	3.25±1.93	3.28±2.01	0.801
“I often feel lonely because I have few close friends with whom to share my concerns”	2.95±1.96	3.29±1.95	3.09±1.87	0.078
“This is frustrating for me as I enjoy mutual conversations with family members and friends”	3.45±2.32	3.47±2.21	3.68±2.22	0.227
“I have not experienced many warm and trusting relationships with others”	2.82±1.87	3.09±1.96	3.13±1.93	0.036
“It is difficult for me to voice my opinions on controversial matters”	2.46±1.83	2.29±1.68	2.46±1.73	0.366

## 4. Discussion

This study assessed the PA levels and well-being of Somali students attending the Somali International University amid the pandemic. This study is among the first to assess the impact of COVID-19 restrictions on PA levels and the well-being of Somali students. To the best of our knowledge, no previous research has been conducted on this subject. The findings of this study provide valuable insights into the effects of the pandemic on the PA patterns and general health of Somali students. The results of the study present intriguing implications from a public health perspective. When taking into consideration the effects of the lockdown that followed the pandemic, authorities throughout the globe used unprecedented containment measures in response to COVID-19's global transmission. The disruption of quarantine forced students to live outside their typical routines and forced them to isolate themselves.

This study revealed that following COVID-19 restrictions, students were physically active, and jogging was the most frequently reported PA. This could be because students had to change their normal schedules and activities or do more PA because of a public health emergency. A global survey indicated that 89% of respondents met the recommended amount of PA in many nations, which is consistent with our study's findings [35]. A previous study reported that students might be highly motivated to engage in PA once they return to their particular campus (Somali International University) [36]. Similarly, 50.8% of upper primary school students met the UK government's moderate and vigorous PA recommendations of 60 minutes per day when they returned to school [37]. Moreover, it was reported that PA levels increased significantly once students returned to school [38]. Lack of PA is a well-known risk factor for cardiovascular disease, which is the world's fourth major cause of death worldwide. Devlin [39] showed that, in Somaliland, a high level of physical inactivity and obesity might become a major public health concern. Obesity (BMI 30 kg/m^2^) was found in 44% of Somali women in Norway and 31% of Somali women in Somaliland, respectively [40].

In the regression analysis, age, gender, academic year, and work status were found to be significant predictors of being physically active after the COVID-19 pandemic. Males and those of younger ages were more likely to be physically active. According to the majority of past research, men are more active than women [41]–[44]. However, research conducted in Brazil utilizing the IPAQ short-form instrument revealed that 41.1% of Brazilians aged 20 and older were inactive [45]. This revealed that the present study seems to be different from that reported in prior studies using other measures of PA, even though it is difficult to compare inactivity prevalence across nations because of variations in survey sampling and assessment methodologies. Also, those in their final year and working remotely were more likely to be physically active. According to data from the Behavioral Risk Factors Surveillance System, a majority of individuals in the United States (54%) fail to meet the recommended levels of PA, which entail at least 30 minutes of moderate-intensity exercise on most days of the week [46].

The COVID-19 pandemic has unexpectedly disrupted the students' lifestyles, despite the level of PA documented in the present research. To limit the spread of the virus, most nations enacted COVID-19 restrictions [47],[48]. Students no longer had access to school-based PA, and schools, sports clubs, and indoor exercise facilities, including swimming pools, were all prohibited. Furthermore, all planned recreational activities were suspended due to lockdown regulations preventing people from gathering even in open spaces. Consequently, recent systematic and scoping reviews revealed significantly lower PA levels compared to pre-pandemic values [49]. In 113 Spanish populations, self-reported PA levels decreased by 91 minutes per day [50]. In the United States, 36% of parents of 211 children aged 5–13 stated that their children had engaged in significantly less PA compared to pre-pandemic levels [51]. These findings are comparable to those of an Italian study that looked at the impact of COVID-19 on PA levels and found a dramatic decrease in PA participation during the COVID-19 pandemic [52]. These results were similar to the findings of Lesser et al [30].

Using the IPAQ-L, it was revealed that in three different universities in Mogadishu, following the relaxation of COVID-19 restrictions, the majority of undergraduate students were physically active [53]. A previous study, which included 2975 respondents and was conducted in Alpine regions (Upper Bavaria, Vorarlberg, Tyrol, South Tyrol, and Trentino) during and after the COVID-19 pandemic, revealed that although COVID-19 restrictions on exercise participation during lockdowns had a short-term negative effect, most respondents eventually returned to their baseline levels of PA following the relaxation of COVID-19 phases [54]. According to a study among 759 respondents, those who had been very active before the lockdown experienced considerably reduced vigorous and moderate intensity PA during and after the lockdown compared to pre-lockdown [55]. A previous study surveyed 2534 students across seven US states during the COVID-19 pandemic. The findings indicated that the pandemic significantly impacted mental health and the level of PA among university students [56].

Public health restrictions have a variety of effects on PA behavior. As low-intensity activities like housekeeping as well as sitting at home and engaging in sedentary activities might be factors that make students inactive, COVID-19 limits may be ascribed to a dramatic change in daily routines and preferences [57]–[59]. Regardless of these facts, when the restrictions were relaxed, the aforementioned facts might explain the finding of our study that age, gender, academic year, and work status were significant predictors of being physically active after the COVID-19 pandemic.

From the study findings, well-being outcomes, namely “not being able to stop or control worrying”, “worrying too much”, “being easily annoyed or irritable about different things”, “feeling afraid as if something awful might happen”, “most people see me as loving and affectionate”, and “not experiencing many warm and trusting relationships with others” had a significant effect on PA participation between those who were more, the same, or less active. It was reported that well-being was strongly linked to PA, and PA was significantly reduced during the COVID-19 pandemic [30]. Lack of motivation might be a factor that can reduce and alter all types of students' PA [60]. Furthermore, Cornine [61] indicated that the constraints on students' PA behavior could be the cause of their fear and anxiety regarding COVID-19, which would impair their mood and make them inactive.

Our study showed that those who exercised outside were substantially more active than students who reported exercising inside. ColleyBushnik and Langlois [62] mentioned that those who exercised outdoors had excellent physical and mental health and comfort compared with those who did not. The majority of students stated that their PA behavior was greatly influenced by their environment, particularly if it was natural, and PA was much higher among those who thought the natural environment was very important. Better mental health has been connected to exposure to nature, with perceived stress acting as a mediator [63],[64]. For nature-related questions, the current study showed that connectedness to nature on the scale had a significant effect on PA participation. This may stem from the impact of COVID-19 precautions on the typical student experience and the heightened anxiety experienced during quarantine [65]–[67].

Limited availability of PA may present obstacles to both the immune system and physical well-being, intensifying pre-existing conditions linked to sedentary lifestyles. Additionally, the absence of opportunities for PA has heightened the stress and anxiety experienced by many individuals facing isolation from their usual social routines. The home quarantine has likely had psychological ramifications, diminishing students' engagement in physical activities, promoting sedentary behaviors, and reducing motivation levels. This is evident in the rising use of mobile devices and the increased average time spent watching television [68]. Maintaining a healthy lifestyle and reducing mortality rates hinge on essential factors such as social support, confidence, and the availability of chances to participate in PA [69].

In contrast to previous studies indicating students' involvement in both indoor and outdoor activities, a higher proportion of students opted for indoor PA during the COVID-19 restrictions [70]. Usually, the option to do PA indoors or outdoors depends on the individual, but the spreading of COVID-19 affected this choice by forcing the closure of athletic and leisure facilities, including sports facilities, forcing students to practice indoor PA [52]. These aspects could be a barrier to engaging in PA in natural environments. The current research revealed that, in the aftermath of the COVID-19 pandemic, certain students found joy in engaging with nature and outdoor activities. The pandemic-induced limitations on exercise and PA faced by numerous students have been identified as contributors to a psychosocial imbalance in mental health. This imbalance stems from the isolation and deprivation of their usual social activities during the pandemic [71].

This research possesses several notable strengths. It marks the inaugural investigation into the PA behavior of Somali students in the aftermath of the restrictions. The study stands out for its utilization of a substantial sample size in assessing PA levels. However, there are certain limitations to consider. There is a potential for selection bias, given that participants were recruited through an online survey shared on personal social media, introducing a degree of self-selection. The inability to gauge anxiety and well-being states before the onset of COVID-19 restrictions presents a challenge in analyzing the impact of public health policies on well-being changes. Furthermore, incorporating assessments of pre-COVID-19 PA behavior would enhance the subjectivity of evaluating alterations in PA levels.

## 5. Conclusion and recommendations

Due to the COVID-19 pandemic, practically all regular human activities were forced to be suspended, preventing students from leaving their houses unless an emergency occurred. These rules impacted students' PA and well-being, notably the stress brought on by lower PA and psychosocial issues like exhaustion. Age, gender, academic year, and work status were identified as factors that significantly influenced PA after the COVID-19 pandemic. Males, younger individuals, and those who engaged in outdoor exercise were more likely to be physically active. Our research demonstrated that participating in outdoor activities and nature brought joy to some students and had a positive impact on their well-being. Physical activity participation was influenced by these well-being outcomes.

This research showed that once the COVID-19 restrictions were relaxed, undergraduate students in Somalia were active. After COVID-19 regulations were relaxed, having such a high level of PA seems beneficial for public health. Consequently, public policies are required to further promote an active lifestyle and prevent sedentary behaviors among Somali undergraduate students. A longitudinal multicenter study is recommended to look at the relationships between PA, mental health, well-being, and quality of life among undergraduate students in Somali universities after the restrictions were relaxed, since the long-term effects of the COVID-19 pandemic remain unknown.

## Use of AI tools declaration

The authors declare they have not used Artificial Intelligence (AI) tools in the creation of this article.
